# Hormonal and Neuromuscular Responses to Mechanical Vibration Applied to Upper Extremity Muscles

**DOI:** 10.1371/journal.pone.0111521

**Published:** 2014-11-04

**Authors:** Riccardo Di Giminiani, Leila Fabiani, Giuliano Baldini, Giovanni Cardelli, Aldo Giovannelli, Jozsef Tihanyi

**Affiliations:** 1 Department of Biotechnological and Applied Clinical Sciences, University of L'Aquila, L'Aquila Italy; 2 Department of Life, Health and Environmental Sciences, University of L'Aquila, L'Aquila, Italy; 3 Laboratory of Chemical-Clinical and Microbiological Analysis, Giulianova Hospital, Teramo, Italy; 4 Department of Biomechanics, Faculty of Physical Education and Sport Sciences, Semmelweis University, Budapest, Hungary; Faculty of Biology, Spain

## Abstract

**Objective:**

To investigate the acute residual hormonal and neuromuscular responses exhibited following a single session of mechanical vibration applied to the upper extremities among different acceleration loads.

**Methods:**

Thirty male students were randomly assigned to a high vibration group (HVG), a low vibration group (LVG), or a control group (CG). A randomized double-blind, controlled-parallel study design was employed. The measurements and interventions were performed at the Laboratory of Biomechanics of the University of L'Aquila. The HVG and LVG participants were exposed to a series of 20 trials ×10 s of synchronous whole-body vibration (WBV) with a 10-s pause between each trial and a 4-min pause after the first 10 trials. The CG participants assumed an isometric push-up position without WBV. The outcome measures were growth hormone (GH), testosterone, maximal voluntary isometric contraction during bench-press, maximal voluntary isometric contraction during handgrip, and electromyography root-mean-square (EMG_rms_) muscle activity (pectoralis major [PM], triceps brachii [TB], anterior deltoid [DE], and flexor carpi radialis [FCR]).

**Results:**

The GH increased significantly over time only in the HVG (P = 0.003). Additionally, the testosterone levels changed significantly over time in the LVG (P = 0.011) and the HVG (P = 0.001). MVC during bench press decreased significantly in the LVG (P = 0.001) and the HVG (P = 0.002). In the HVG, the EMG_rms_ decreased significantly in the TB (P = 0.006) muscle. In the LVG, the EMG_rms_ decreased significantly in the DE (P = 0.009) and FCR (P = 0.006) muscles.

**Conclusion:**

Synchronous WBV acutely increased GH and testosterone serum concentrations and decreased the MVC and their respective maximal EMG_rms_ activities, which indicated a possible central fatigue effect. Interestingly, only the GH response was dependent on the acceleration with respect to the subjects' responsiveness.

## Introduction

Although whole-body vibration (WBV) is receiving much interest as an alternative exercise modality in sport and rehabilitation physiology, its neuromuscular and endocrine system effects have yielded conflicting results [1], [2], [3], [4], [5] and are not yet completely understood [6], [7]. Additionally, the acute hormonal responses to WBV exercise have not been extensively studied.

In the extensive resistance training literature, a bout of resistance exercise produces acute hormonal environment changes that are largely dependent on the resistance exercise parameters [8], [9]. The increases in the concentrations of hormones, such as testosterone and GH, are proportional to the recruited muscle volume relative to the exercise intensity [9]. Consequently, training principles have been constructed to maximize the post-exercise increase in these hormones based on the hypothesis that exercise-induced systemic hormone increases will influence or optimize functional performance gains in whole body skeletal muscle mass and strength adaptations [10], [11]. In this regard, the gravitational load has been increased to superimpose an additional external load during WBV to better stimulate the neuroendocrine system and maximize the post-exercise increases in testosterone and GH hormone concentrations [5], [12]. However, the gravitational load was not defined with respect to the WBV responsiveness of the subjects. Therefore, an additive pulsatile GH secretion effect was attained in healthy females [5] by combining WBV with an external load, but this effect was not detected in severely obese females [12].

To date, no previous studies of the acute hormonal WBV response have examined the optimal combination of the acceleration load (hyper-gravity condition) and the posture assumed on the vibrating plate in an attempt to recruit a greater number of muscle fibers that would enable a greater hormone-tissue interaction within a relatively larger total muscle volume [9]. During WBV, as the vibrating platform movement is sinusoidal, the acceleration load is quantified by calculating the acceleration transmitted to the body according to the following equation a = ***A***(2π*f*)^2^ [7]. In this equation, A represents the oscillation amplitude and f represents the frequency. The changes in the amplitude and/or the frequency therefore determine the acceleration and vibrational magnitude changes that are transmitted to the body.

Regarding posture or exercise on the vibrating plate, push-ups (isometric) can provide an adequate stimulus to acutely increase testosterone concentrations [13]. This basic exercise could represent a potent physiological neuroendocrine system stressor because it involves a large muscle volume (trunk + upper arm + lower arm) [14]. Notably, many muscles of this volume require considerable muscle activation relative to their maximal isometric values [15], [16]. Additionally, by comparing the standing (half squat) and horizontal positions (similar to a push-up), the trunk muscles are more strongly activated in the horizontal position during WBV [17].

### Hypotheses

Based on these previous findings, we hypothesized the following:

Vibration applied to the upper body induces pronounced hormonal and neuromuscular responses, and the magnitudes of the hormonal and neuromuscular responses are dependent on the acceleration load relative to the subjects' responsiveness.

### Aim

The purpose of the present study was to determine the acute residual hormonal (GH and testosterone) and neuromuscular (maximal voluntary contraction, mechanical power, and EMG activity) responses following a single WBV bout superimposed on an isometric push-up using two different acceleration loads. The acceleration loads were individualized relative to the participants' responsiveness by monitoring the EMG muscle responses (PM, TB, DE, and FCR) of the participants [18], [19], [20].

## Methods

### Study design and participants

A randomized double-blind, controlled-parallel trial design was used for this study. The participants were blinded to the study hypotheses, and the assessors were blinded to the group allocation. Among two hundred students within a sport sciences department, 30 male students elected to participate in this experiment after being informed of the procedures and potential risks involved in the study. The exclusion criteria included a history of back pain, acute inflammation in the pelvis and/or lower extremities, acute thrombosis, tumors, recent fractures, recent implants, gallstones, kidney or bladder stones, any disease of the spine, peripheral vascular disease, and severe delayed trunk and arm muscle soreness onset. All participants gave written informed consent. One investigator in the study (author's last name) enrolled the participants. The sample size for the primary outcomes was computed a priori based on the α (0.05), power (1-β; 0.90) and effect size values of previous studies [1], [5], [13] by means of a statistical software for power analysis (G*Power 3.1.9, Heinrich Heine-Dusseldorf University). The sample size computation was performed while taking into account the study design and using both parametric and nonparametric procedures. The participants were randomly assigned to a high-vibration group (HVG) (age: 25.0±0.9 years; weight: 73.0±2.9 kg; height: 177±2.1 cm; body mass index: 23.2±0.7 kg m^−2^; lean body mass: 57.8±1.8 kg), a low-vibration group (LVG) (age: 26.0±1.5 years; weight: 76.0±2.8 kg; height: 178±2.3 cm; body mass index: 23.7±0.6 kg m^−2^; lean body mass: 59.6±1.8 kg) or a control group (CG) (age: 24.0±0.8 years; weight: 75.7±2.9 kg; height: 178±2.1 cm; body mass index: 23.7±0.6 kg m^−2^; lean body mass: 59.3±1.82 kg). The lean body mass values were estimated using a semi-mechanistic model [21]. The random allocation sequence was generated using an algorithm with equal allocation ratios, random sorting, and a maximum allowable deviation of 20%. The requirements for this algorithm were that each group had exactly 10 subjects and the maximum % deviation was no larger than 20% of the target sample size. The sequences were generated using statistical software (Pass 13-NCSS, LLC Kaysville, Utah 84037, USA). The Ethics Committee of the University of L'Aquila approved the study conducted between May and July 2012. The measurements and interventions were performed at the Laboratory of Biomechanics of the University of L'Aquila. The participants were healthy and recreationally active and all within similar fitness levels to reduce the possible influence on the hormonal serum changes. Similarly, the participants had to eliminate strength and endurance exercises from their training during the period of the experiment. Therefore, the training regimen was composed of only the physical activities (no strenuous exercises) performed in their sport sciences department. No participants reported drug or nutritional supplement ingestion known to interfere with GH and/or testosterone secretion.

### Experimental day procedures

**Figure 1:**
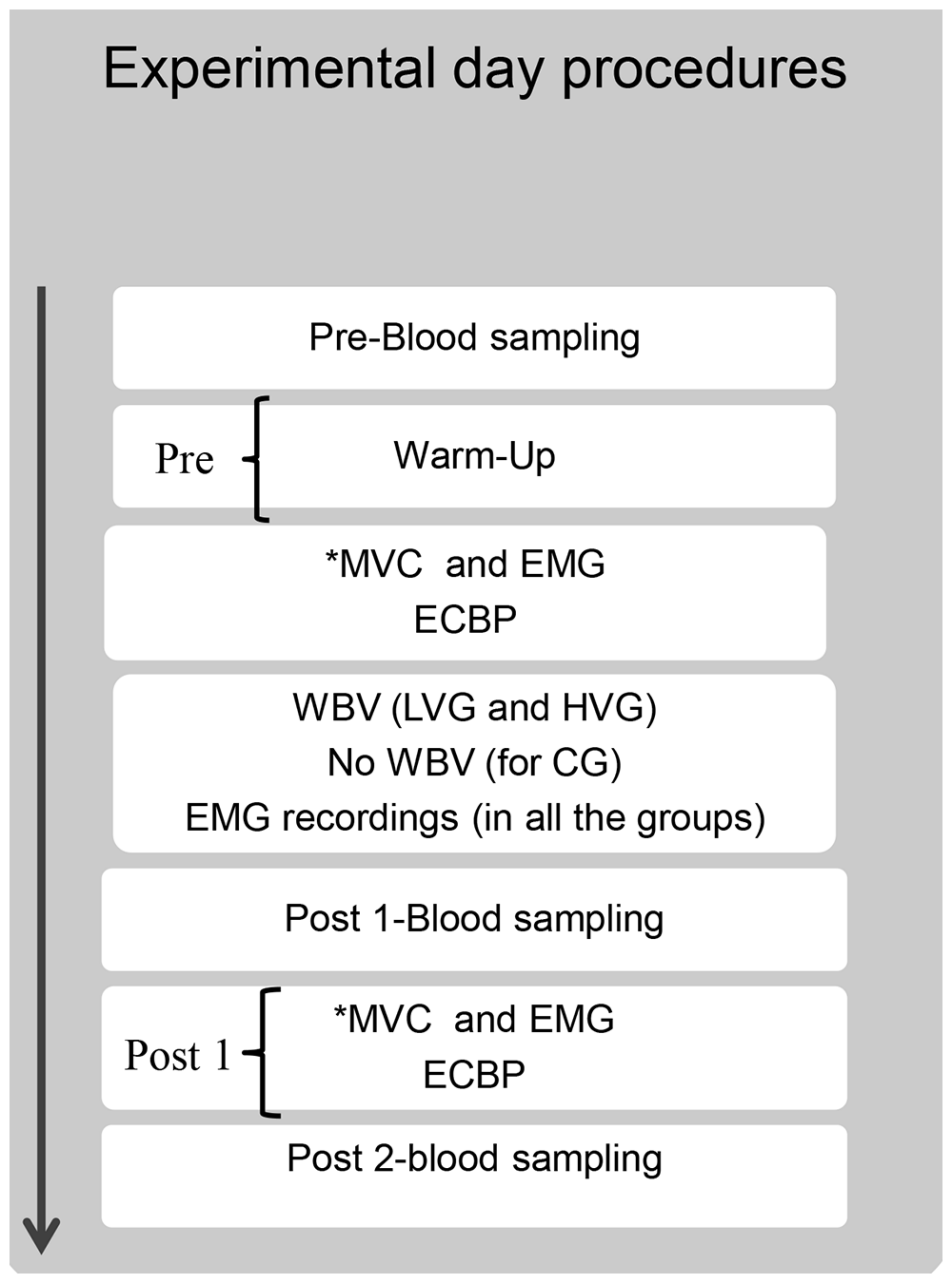
Experimental protocol of the study design. ^*^Maximal Voluntary Isometric Contraction was performed during bench press and handgrip tests. HVG-High Vibration Group; LVG-Low Vibration Group; CG-Control Group; ECBP-Eccentric-Concentric Bench Press; Pre: immediately before the vibrational interventions; post 1-immediately after the vibrational interventions; post 2-1 h after the end of the vibrational interventions.

For each participant, the measurements were performed over a 2-day period to reduce any potential boredom and fatigue effects. Each test session began with a 15-min warm-up (6 min of treadmill running at a speed of 6 km h^−1^; 4 min of stretching; conventional trunk, arm, and shoulder joint exercises; and five low-speed push-ups). The participants were introduced to the equipment and procedures during a familiarization period. On the first day, each participant performed maximal voluntary isometric contractions (MVC) during bench press and handgrip tests. The EMG activity, which was synchronized to these strength measurements, was also recorded. Then, the EMG activity was recorded during WBV to estimate the optimal acceleration load in relation to subject responsiveness. All measurements taken on the first day were thus performed to determine the vibrational load, which was normalized to each subject's MVCs. On the second day (Figure 1), baseline measurements (Pre) were taken, including blood sampling for hormones analyses, MVC synchronized to EMG activities and mechanical variables during the eccentric-concentric bench press test. Next, each subject was provided a treatment (specific to his assigned experimental group, see below), and EMG activities were recorded during the interventions. The measurements were repeated immediately after the treatments ended (Post1) and 1 hour after the treatment ended (Post 2). Blood was sampled also during Post 2 due to hormonal profile changes observed in a previous pilot study All measurements were performed at consistent times of day (4.00–8.00 PM) to reduce the potential diurnal variation effect on hormone secretion and neuromuscular variables [22].

#### Blood collection and hormonal analyses

On the second experimental day, the subjects visited the laboratory and rested for 30 min prior to the first blood collection. Blood samples were drawn into vacutainers from the antecubital forearm vein using a 20-gauge needle (without additives) for the total serum testosterone and GH concentration measurements. All samples were obtained with the subjects placed in a seated position in a climate-controlled environment (21°C). Whole blood was stored at 18–25°C until a clot formed (15–45 min); then, the blood was centrifuged at 3,000 rpm for 5 min to obtain the serum specimen for future assays. The serum samples were stored at 2–8°C and analyzed within 24 h. All samples were assayed in duplicate and were decoded only after the analyses were completed. The total testosterone and GH concentrations were determined using commercially available enzyme immunoassay kits (ST AIA-PACK 2000, Tosoh Corporation, Tokyo, Japan) according to the manufacturer's instructions. The testosterone and GH assay sensitivities were 0.07 ng/ml. The intra- and inter-assay variation coefficients were 3.3% and 3.7% for testosterone and 1.4% and 3.3% for GH, respectively.

#### Maximal voluntary isometric contraction

MVC was performed using a custom exercise rack in which the subjects were positioned in a supine isometric bench press position [23]. MVC was also measured on the dominant hand using a custom handgrip attached to a strain gauge (Ergotest-Innovation, Porsgrunn, Norway). The handgrip measurements were performed using a standardized method [24]. During MVC, following a 2-min warm-up period, the subjects performed a maximum of three attempts without a time constraint, and the final average value was calculated (Figure S1). Each warm-up and maximal attempt was separated by 1–2 min of rest. The participants were instructed to contract their muscles as hard and fast as possible, and strong verbal encouragement was provided during each MVC attempt [25]. The intra-day MVC during bench press and handgrip reliabilities were 0.96 for both tests, whereas the inter-day reliabilities were 0.82 and 0.88, respectively.

#### Eccentric-concentric bench press

The participants performed an eccentric-concentric bench press exercise at a maximal speed. The external load applied to the bar was equal to 35% of the MVC. During the movement, the bar was mechanically linked to a linear encoder (Ergotest-Innovation, Porsgrunn, Norway). Thus, it was possible to calculate the power that corresponded to the load displacement [26]. Strong verbal encouragement was provided to the subjects, and three attempts were performed. The mean of the three attempts was calculated for further analyses. The intra-day and inter-day displacement reliabilities during the eccentric-concentric bench press exercise were 0.94 and 0.91, respectively.

#### EMG recordings

The EMG surface activity was recorded using bipolar disc electrodes (Blue-Sensor Ambu Ag/AgCl, type NF-00-S, dimensions, 44.3×22×22 mm), an amplifier (gain setting, 100 Hz; input impedance, 1000; 2 GΩ common mode rejection rate, 100 dB; input noise level [1 kHz band], 3 µVcc), and a Butterworth band-pass filter (3-dB low cut-off frequency, 8 Hz; 3-dB high cut-off frequency, 1200 Hz) fixed over the muscles. The electrodes were placed according to the surface EMG for non-invasive assessment of muscles (SENIAM) recommendations [27] on the dominant side of the body. To maintain consistent electrode positioning across the inter-day EMG recordings, the subjects' skin positions were marked with indelible ink.

The EMGrms was recorded for the PM, DE, and TB muscles during MVC at bench press and for the FCR muscle during MVC at handgrip (Musclelab V8.10, Ergotest Innovation, Porsgrunn, Norway). The force times and the EMG time histories were synchronized with respect to time and during the off-line analyses, the maximum force values and the EMGs (400 ms window) were considered in the analysis (Figure S1). The intra-day and inter-day EMG reliabilities were 0.97 and 0.97 for the PM muscle, 0.96 and 0.85 for the DE muscle, 0.95 and 0.85 for the TB muscle, and 0.96 and 0.68 for the FCR muscle, respectively. The EMGrms was also recorded during the treatments (in each trial) for all of the subjects. Delta (Δ, %) was calculated as the difference between the first and last trials according to the following formula: [(last trial – first trial)/first trial]*100. The intra-day reliabilities were 0.95, 0.92, 0.94, and 0.80 for the PM, DE, TB, and FCR muscles, respectively.

### Interventions

The participants postured themselves in the push-up position on the vibration device (Nemes-Lsb, Bosco-System, Rome, Italy) while flexing the elbow at approximately 90° (Figure S2). The HVG and LVG participants were exposed to a series of 20 trials ×10-s synchronous WBV with a 10-s pause between each trial and a 4-min pause after the first 10 trials. The CG participants assumed an isometric push-up position without WBV. The HVG participants were exposed to synchronous whole-body vibration at 40 Hz (the displacements was ∼0.9 mm and the acceleration load ranged from 2.88 to 5.72 *g*), whereas the LVG participants were exposed to 20 Hz (∼0.2 mm and the acceleration ranged from 0.12 to 0.36 *g*) (Materials S1). The detailed characteristics of the interventions in the three groups and the protocol used to detect the acceleration loads are reported in the supplemental section (Table S1).

### Statistical analyses

Because Shapiro-Wilk's W test for normality revealed that the data were not normally distributed, the analyses were performed using non-parametric statistical tests. The effect of WBV on the neuromuscular and hormonal variables was assessed over time and in each group using Friedman's test. The Wilcoxon test was used to detect differences based on within-group comparisons and the Bonferroni correction was used to adjust the P-values according to the number of comparisons that were performed. The between-group comparisons were performed using the Kruskal-Wallis test. Cronbach's alpha coefficient was used to determine the reliability between the participants. The analyses were performed using XLSTAT 13.02 statistical software (Addinsoft, SARL, NY, USA). Statistical significance was set at P<0.05, and the meaningfulness of the significant outcomes was estimated by using the effect size (ES) of Cohen.

## Results

The results of 30 participants were analyzed during the test sessions. The participants did not report side-effects or muscle-tendon injuries, and none of the baseline measurements (descriptive characteristics and experimental data) were significant among the groups (P>0.05).

### Hormonal responses

**Figure 2:**
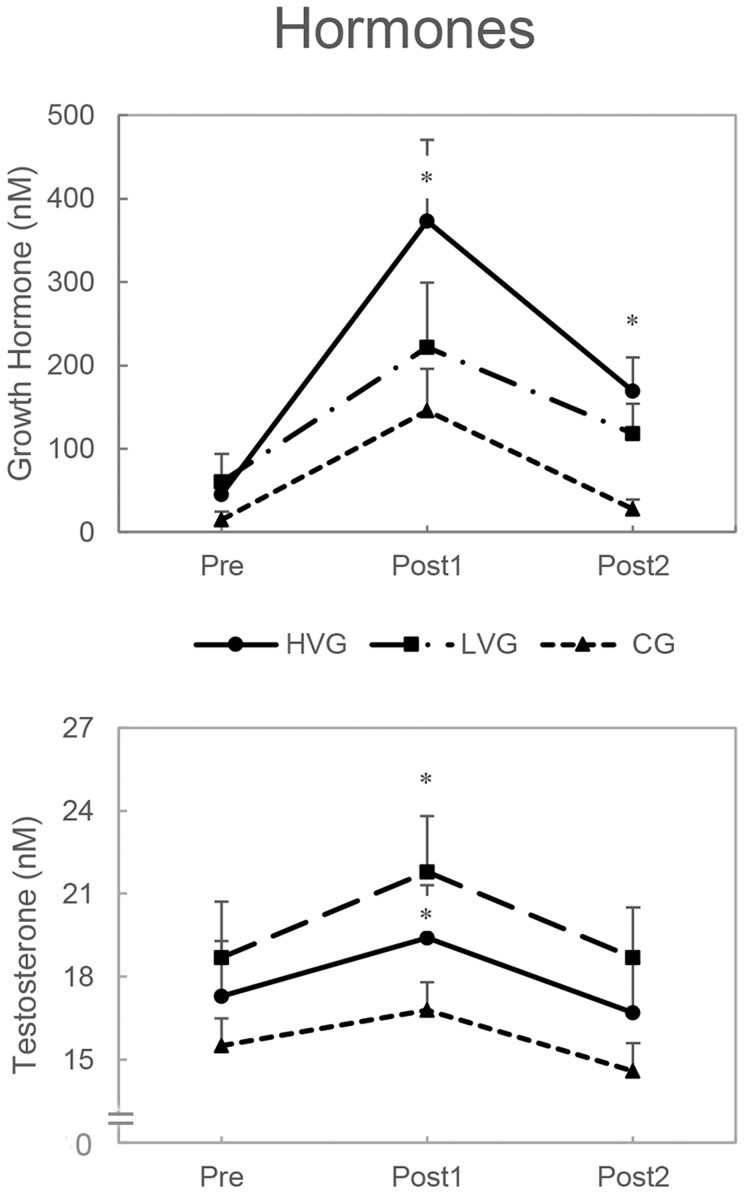
Absolute changes in the hormone concentrations (mean, SE) measured immediately before (pre), immediately after (post1), and 1 h after the end (post2) of the vibrational interventions. *Significant pre-post effect within group (P<0.05). No significant difference between the HVG and the LVG (P>0.05).

The GH response significantly increased over time in the HVG (P = 0.003) but not in the CG or the LVG. Additionally, comparative analyses revealed significant changes in the HVG at Post1 (P = 0.001, ES = 1.05) and Post2 (P = 0.014, ES = 0.92) (Figure 2). A significant testosterone response over time was also detected in the LVG (P = 0.011) and the HVG (P = 0.001) but not in the CG. The differences were located at Post1 in LVG (P = 0.014, ES = 1.07) and HVG (P = 0.001, ES = 1.53); however, the differences between the two experimental groups were not statistically significant. No significant changes were observed for any group at Post2 (Figure 2).

### Maximal voluntary isometric contraction and eccentric-concentric bench press

**Figure 3:**
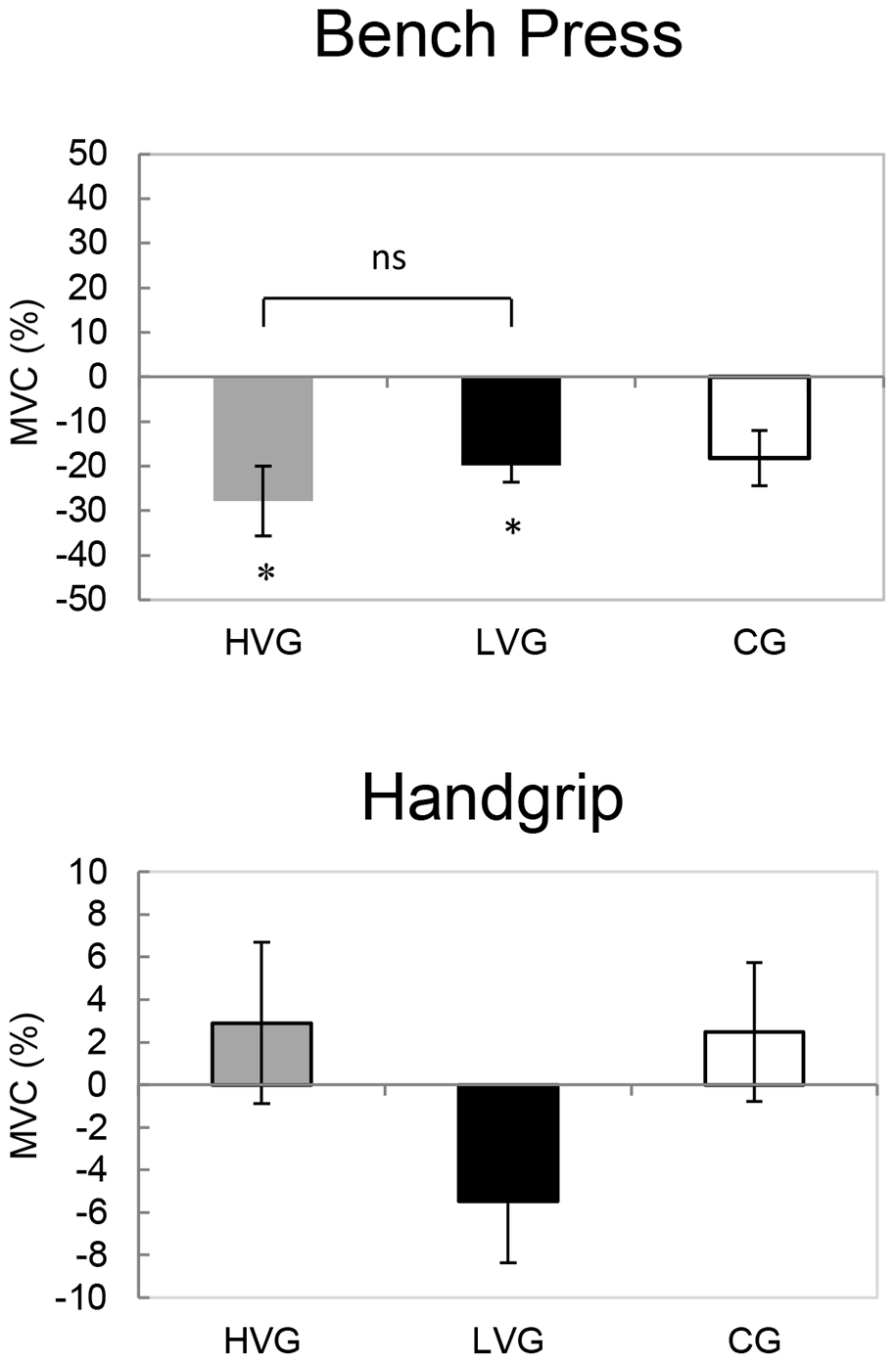
The relative changes (mean, SE) in the maximal voluntary isometric contraction during bench press and during handgrip (pre-post1) are shown for the CG, the LVG, and the HVG. Immediately before (pre) and after (post1) the vibrational interventions. ^*^Significant pre-post 1 effect within a group (P<0.05); ns, no significant differences between the groups (P>0.05).

**Figure 4:**
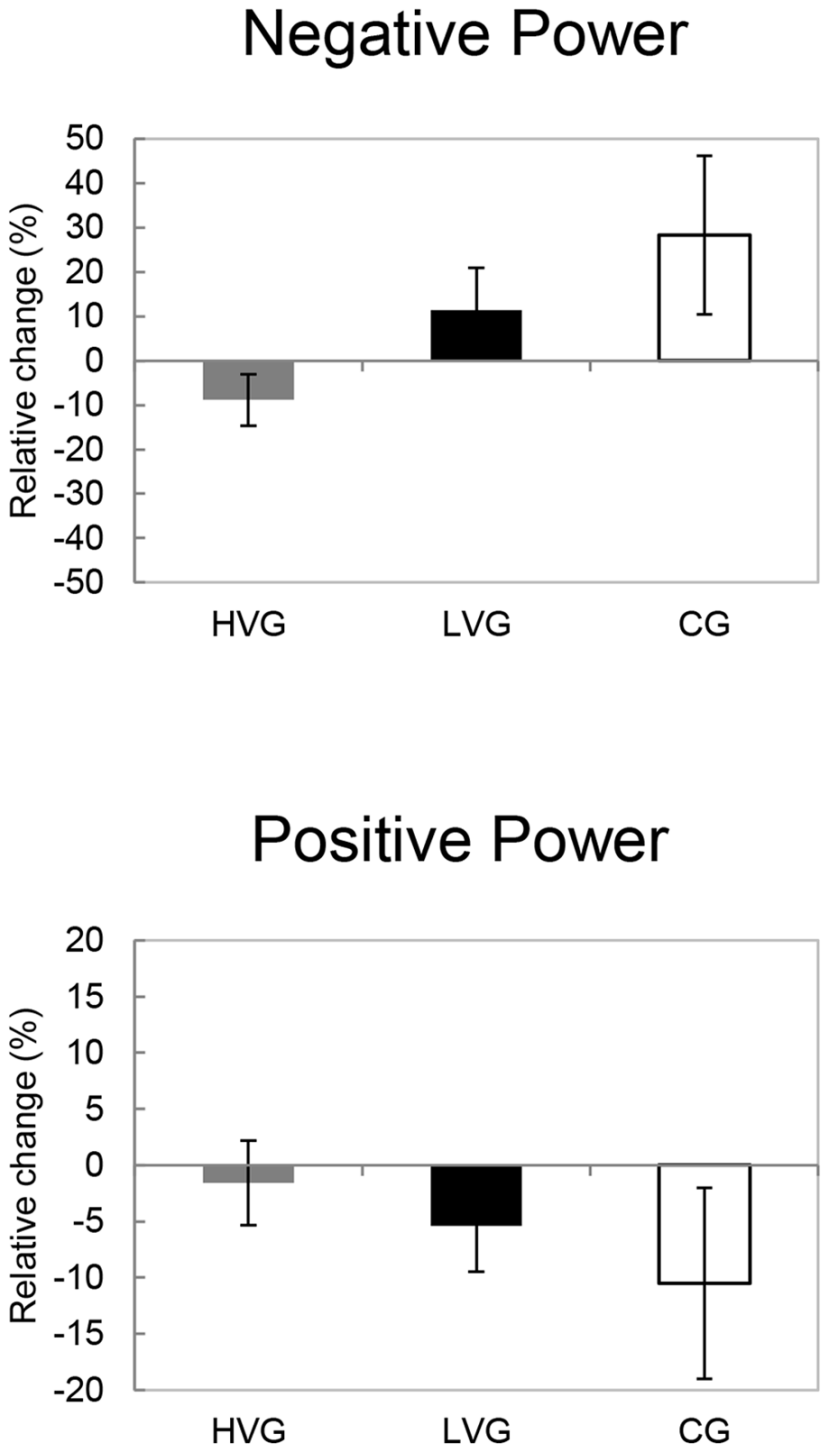
The relative changes (mean, SE) in the negative and positive power during eccentric-concentric bench press exercise (pre-post1) are shown for the CG, the LVG, and the HVG. The differences were not significant (P>0.05).

MVC during bench press decreased significantly in the LVG (P = 0.001, ES = 1.65) and the HVG (P = 0.002, ES = 1.32), whereas there was no significant decrease in the CG; however, no significant differences were detected between the LVG and the HVG. MVC during handgrip did not change significantly in any group (Figure 3). The effects of WBV on the negative and positive power during eccentric-concentric bench press exercise did not significantly change in any group (Figure 4).

### EMG activity recorded during maximal voluntary isometric contraction

**Figure 5:**
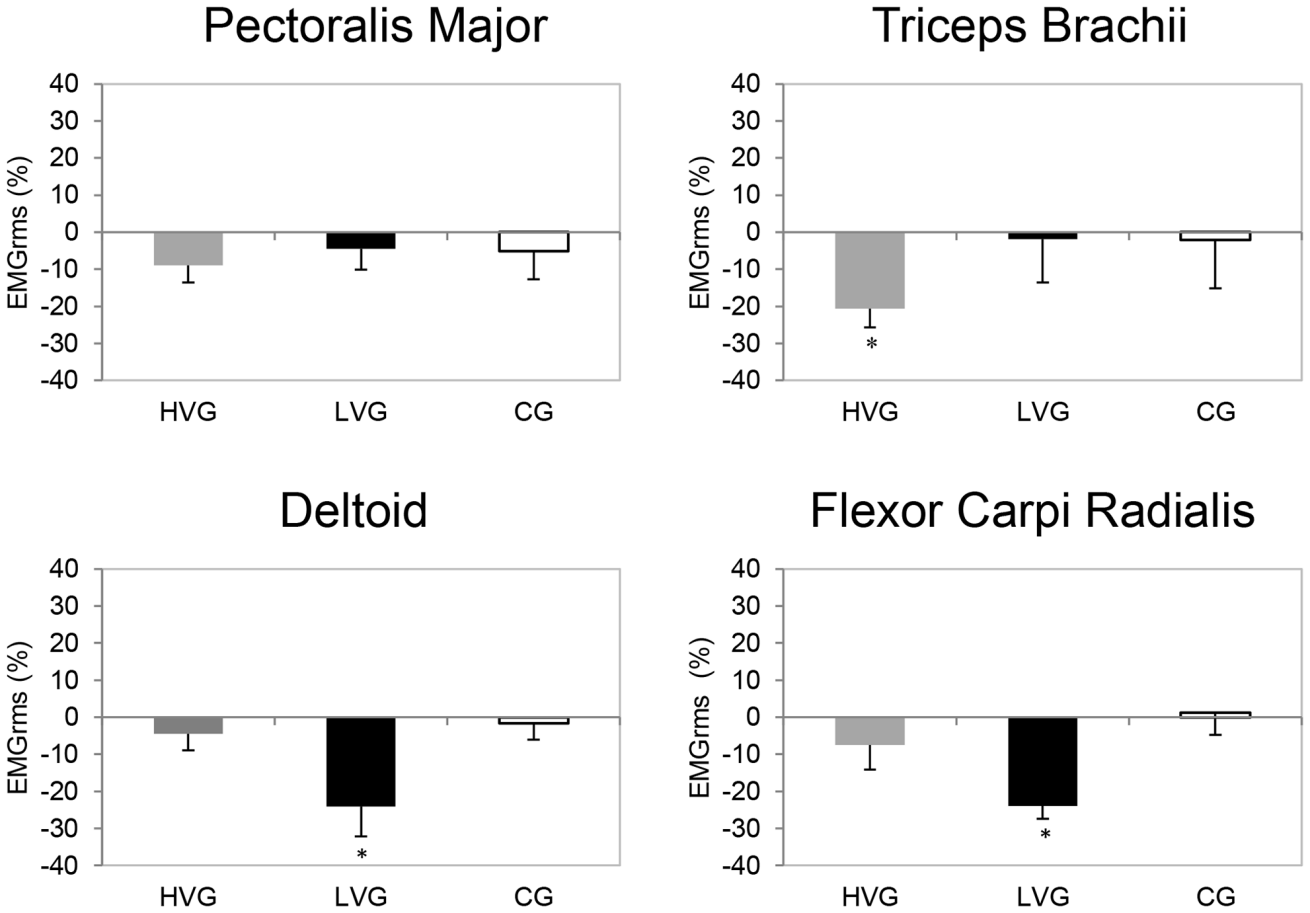
Relative changes (mean, SE) in the EMG_rms_ (recorded during MVC) are shown for the CG, the LVG, and the HVG. ^*^Significant time effect (pre-post1) within a group (P<0.05).

In the PM muscle, the EMG responses did not decrease significantly in any group. The TB muscle EMG decreased significantly in the HVG (P = 0.006, ES = 1.17) but not in the LVG or the CG. Conversely, the DE muscle EMG responses significantly decreased in the LVG (P = 0.009; ES = 0.56); however, no significant changes were detected in the CG or the HVG. Similar to the DE muscle, the FCR EMG responses to WBV were significantly different in the LVG (P = 0.006; ES = 1.41) but not in the CG or the HVG (Figure 5).

### EMG activity during interventions

**Figure 6:**
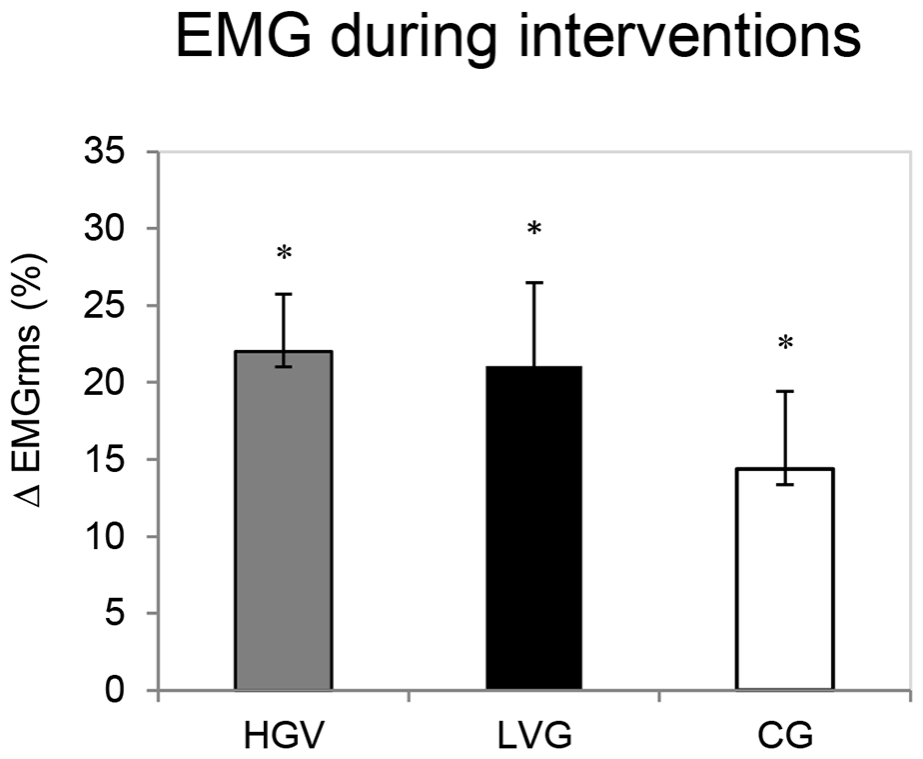
EMG activity during vibrational interventions. The Δ EMG_rms_ (mean, SE) (normalized to MVC) of all muscles was averaged within each group during the vibrational interventions. ^*^Significant time effect within a group (P<0.05). Delta (Δ, %) was calculated as the difference between the first and the last trials according to the following function: [(last trial – first trial)/first trial]*100.

Because the pattern did not change among the muscles during the several repeated trials, the average EMG activity of all muscles within each group was analyzed (Figure 6). The EMG_rms_ increased progressively in all of the groups, and Δ was significantly different in the LVG (P = 0.009, ES = 1.78), the HVG (P = 0.006, ES = 1.88), and the CG (P = 0.014, ES = 0.90). No significant differences between the groups were observed.

## Discussion

The primary finding of the present study includes a significant increase in GH immediately following the end of exercise (+380-fold) only in the case of the high acceleration load. Additionally, the GH concentration remained elevated until 1 h after the end of WBV exercise (+180-fold). In contrast, the testosterone concentration was elevated for both the 2.88 *g* (+15%, HVG) and 0.12 *g* (+21%, LVG) acceleration loads.

It is well known that plasma GH concentrations increase during exercise and that the magnitude of this response appears to be dependent on the exercise selected and the volume of muscle mass recruited, the muscle contraction type, the exercise intensity, the exercise duration, the rest interval between sets, and the total magnitude of work performed [9]. In this study, the GH response magnitude was associated with the peak exercise intensity, as all other variables were equal between the groups. The WBV exercise intensity in the HVG, which was estimated based on the EMGrms activity, produced the greatest muscle activation during the vibrational conditions. Therefore, upon exercise onset (WBV and isometric push-ups), the signals that arose in the central areas of the brain (central command) activated the motor cortex to induce muscle contractions in a potentially parallel manner and activated more endocrine centers to cause increased GH release [28]. Thus, the GH response in our study appears to be proportional to the recruited muscle volume relative to the exercise intensity, similar to resistance exercise [9]. Additionally, the increase in EMG activity recorded toward the end of each repetition (Delta) could cause an increase in reflex activation [29], [30], which indicates that the “central command” can be intensified via feed-back signaling from the exercising muscles mediated by the afferent nerves as neural reflex mechanisms [9], [28]. Concisely, it may be possible for higher brain centers (i.e., the motor cortex) to play an active role in regulating GH secretion during WBV and this regulatory mechanism may be sensitive to specific peripheral neural mechanisms.

With respect to the testosterone response, it is unclear why this response was not dependent on the WBV intensity. Traditionally, the physiological function of testosterone in skeletal muscle tissue is the maintenance of an increase in skeletal muscle mass (hypertrophy) via genomic (long-term, transcriptional) mechanisms and subsequent indirect increases in muscle strength [8], [9]. However, steroid hormones, including testosterone, have been demonstrated to elicit rapid responses (within seconds to minutes) in rat muscle fibers [31] via non-genomic mechanisms (short-term, non-transcriptional). According to this perspective, given that testosterone regulates a wide biological body function spectrum with respect to environmental changes, the acute control of testosterone levels might be more complex (by the system) than the simple and direct regulation of testosterone levels by WBV exercise intensity.

The WBV-induced suppression of MVC and EMG activity could indicate the presence of significant ‘central fatigue’. However, the increase in EMG activity during the treatments (delta) suggests an increase in reflex activation in which the motor units are recruited at the lowest force thresholds (compared with voluntary activation) to compensate for a reduced descending drive in an attempt to maintain the force output during fatigue [29]. Additionally, the selective attenuation of the maximal muscle EMG activity following WBV is dependent on the acceleration load, which suggests that the afferent input could be dependent on the activated muscle fiber types [30], [32], the different muscle spindle responses to muscle vibration, and the specific sensory receptor population [33], [34]. Neuromuscular propagation failure, muscle-tendon unit compliance, motor cortex excitability, and muscle fatigue status may be related to WBV-induced suppression of the maximal voluntary contraction [32].

Muscle fatigue is characterized by not only a loss of force generation capacity but also a slowing of the muscle contractile speed. Indeed, loss of power is a major consequence of muscle fatigue due to changes in three muscle properties: a decrease in the isometric force, a slowing of the maximal velocity, and an increase in the force–velocity function slope [35]. The metabolic mechanisms underlying these changes have not been identified, but it has been speculated that a decrease in activating calcium might trigger the loss of power during fatigue [35]. In this study, the negative and positive power measured during the eccentric-concentric bench press exercise remained unchanged in all three groups after WBV. Therefore, it appears most likely that WBV-induced suppression of the MVC is not caused by changes in the muscle or tendon mechanical and metabolic properties. Based on the results of prior non-genomic testosterone analyses and the absence of a significant loss of power in our study, we cannot exclude the possibility of protective effects of testosterone against skeletal muscle fatigue as suggested by Bosco et al. [1], [36].

Although histological alterations provide a direct evidence for muscle damage, often, this damage is assessed indirectly by increases in muscle proteins in the blood (e.g., creatine kinase: CK), ratings of delayed onset muscle soreness, and decreases in maximal voluntary contraction and range of motion [37]. Therefore, in the present study, the significant MVC decrease could also indicate the presence of muscle damage because MVC peak force measures have been considered to be valid markers of muscle damage [37]. In this connection, a recent study of Hazell et al. [38] has demonstrated that synchronous whole-body vibration superimposed on moderate exercise determines significant increases in interleukins (IL-1b, IL-6, IL-10), indicating the effects on muscle damage and inflammation, even if the reported value in IL-6 (∼2–3 pg•ml^−1^) is less than that reported after a traditional resistance training session (∼5–6 pg•ml^−1^) [39].

In our study, the combination of vibration plus posture assumed on the vibrating plate produced an intense exercise during the vibrational interventions, particularly within the high vibration group. In fact, the pectoralis major muscle activation approached the maximal values recorded during the maximal voluntary isometric contraction (bench press). Therefore, during the vibration training (toward the end of the treatment-the last two repetitions) the participants were not be able to sustain the workload and dropped on the plate. As previously described, the fatiguing task is speculated to cause a myofibril disruption that triggers an inflammatory response increasing the circulation of cytokines and hormones that promote muscle tissue remodeling [11], [39], [40]. However, a conclusive scheme cannot be depicted because pro-inflammatory cytokines (i.e., IL-6) and other systemic muscle proteins (CK) were not measured.

## Limitations

### GH pulsatile characteristics

The major point of concern in our study is the pulsatile characteristics of the GH response. These pulsatile events in healthy young men are regular and occur during the night [41]. All measurements were performed consistently each day (4.00–8.00 PM). Because two significant peaks relative to the baseline value (Post1 and Post2) were detected in only one experimental group, the possibility of individual diurnal variation effects on the GH concentration in the HVG can be excluded [22].

### Plasma volume

Plasma volume changes could influence the hormone concentrations. Furthermore, there are no studies in the literature that have addressed this issue during WBV. However, plasma volume can be influenced by prolonged strenuous endurance exercise (e.g., cycling, ultra-marathon) [42] and/or by prolonged posturing in the horizontal position (for 5 h after intense aerobic exercise performed at 85% of the peak oxygen consumption rate) [43]. Additionally, acute resistance exercise (three sets of 5–7 repetitions of six exercises at an intensity corresponding to 80% of the one-repetition maximum, 1-RM) can decrease the plasma volume by approximately 10% [44]. Therefore, these relative changes cannot explain the large increase in the hormone concentration, particularly GH (+380-fold).

### Generalization of the results

Because our study subjects were healthy, male, recreationally active participants, our results cannot be generalized to other populations. A specific type of participants was recruited to reduce the influence of potentially confounding variables (sex, age, and level of training).

## Conclusions

### EMG activity and acceleration load

The data presented show that EMG monitoring during different acceleration loads can represent an appropriate method of assessing the optimal magnitudes to acutely maximize the GH and testosterone serum concentrations when applying vibration to the upper extremities in a similar manner as resistance exercises [8], [9].

### Fatigue

Synchronous WBV-induced fatigue decreases the MVC and the related EMG activity, indicating a fatigue effect that appears to be related to “central” [29], [45] rather than “peripheral” factors [46].

## Supporting Information

Figure S1 **Representative MVC and rectified EMG data for the PM, DE, TB, and FCR muscles during the isometric bench press and handgrip tests performed without time constraint by 1 of the subjects.** The shaded area represents a 400 ms window around the force peak, which was used to compute the EMGrms values for the selected muscles. (Bench press) The force plate was set to 0 when the bench stood on its own without the subject. BW, body weight.
(TIF)

Figure S2 a) Position assumed by the subjects on the vibrating platform (A). b) The peak acceleration and vibrating plate displacement values were measured as the vibration frequency was increased by 5 Hz every 5 s from 20 to 55 Hz. The acceleration load values ranged from 0.1 to 5.7 *g* (expressed as a multiple of standard gravity, where 1 *g* is equal to 9.81 m·s^−2^). The displacement was calculated and ranged from 0.15 to 1.21 mm. c) The normalised EMG_rms_ values (mean and SE) of the participants, which were recorded during WBV at different acceleration loads. The circles indicate the acceleration loads selected for the LVG (0.12 *g*) and the HVG (2.88 *g*).
(TIF)

Table S1 **Characteristics of the vibrational intervention.**
(DOCX)

Materials S1 **Experimental protocol for acceleration load determinations.**
(DOCX)
