# Supplementary figures and images for: Hormonal and Neuromuscular Responses to Mechanical Vibration Applied to Upper Extremity Muscles
Source: PLoS One. 2014 Nov 4;9(11):e111521. doi: 10.1371/journal.pone.0111521 (PMC4219718; doi:10.1371/journal.pone.0111521)

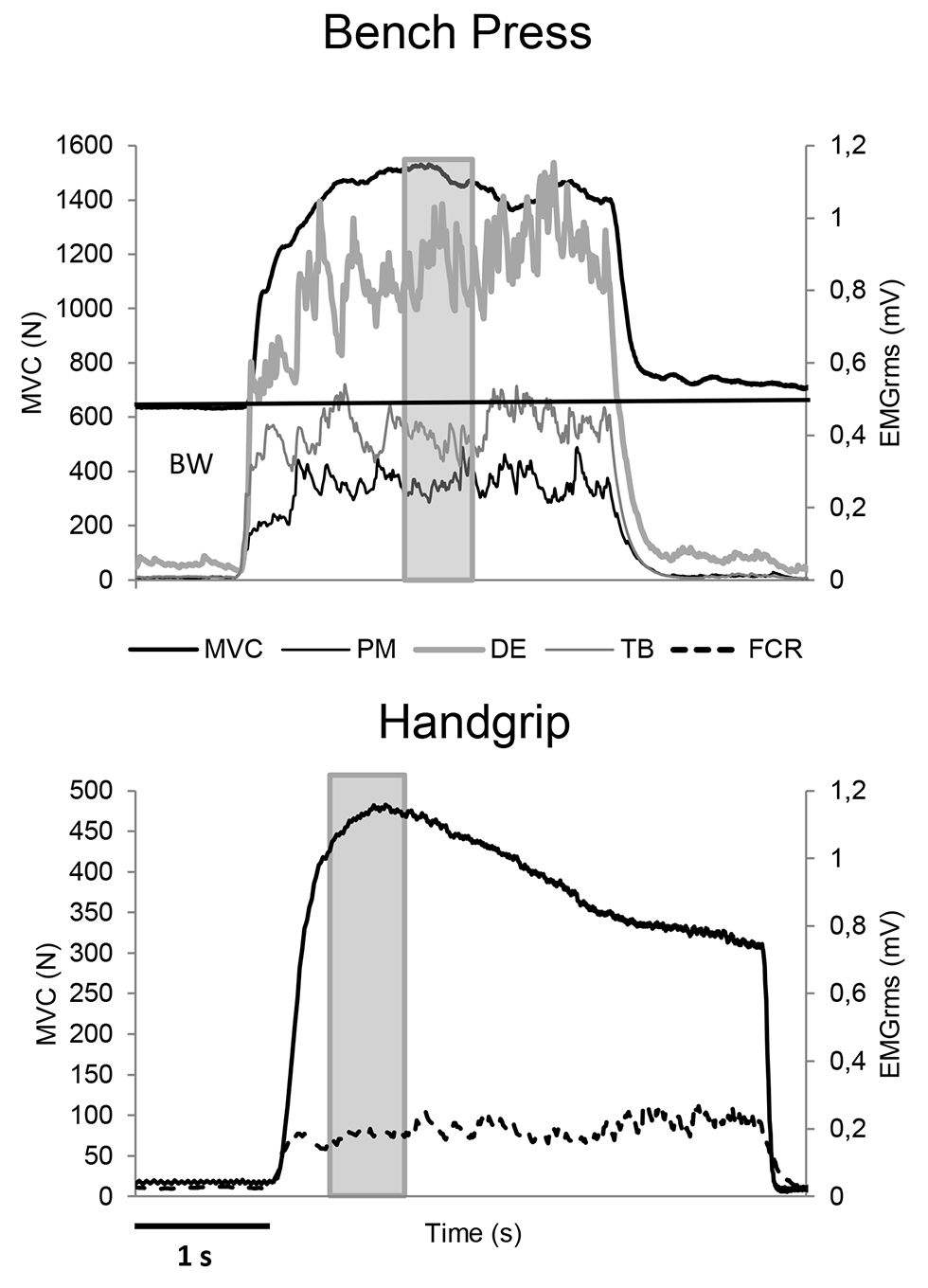

Supplement: Figure S1 — Representative MVC and rectified EMG data for the PM, DE, TB, and FCR muscles during the isometric bench press and handgrip tests performed without time constraint by 1 of the subjects. The shaded area represents a 400 ms window around the force peak, which was used to compute the EMGrms values for the selected muscles. (Bench press) The force plate was set to 0 when the bench stood on its own without the subject. BW, body weight. (TIF) [file pone.0111521.s001.tif]

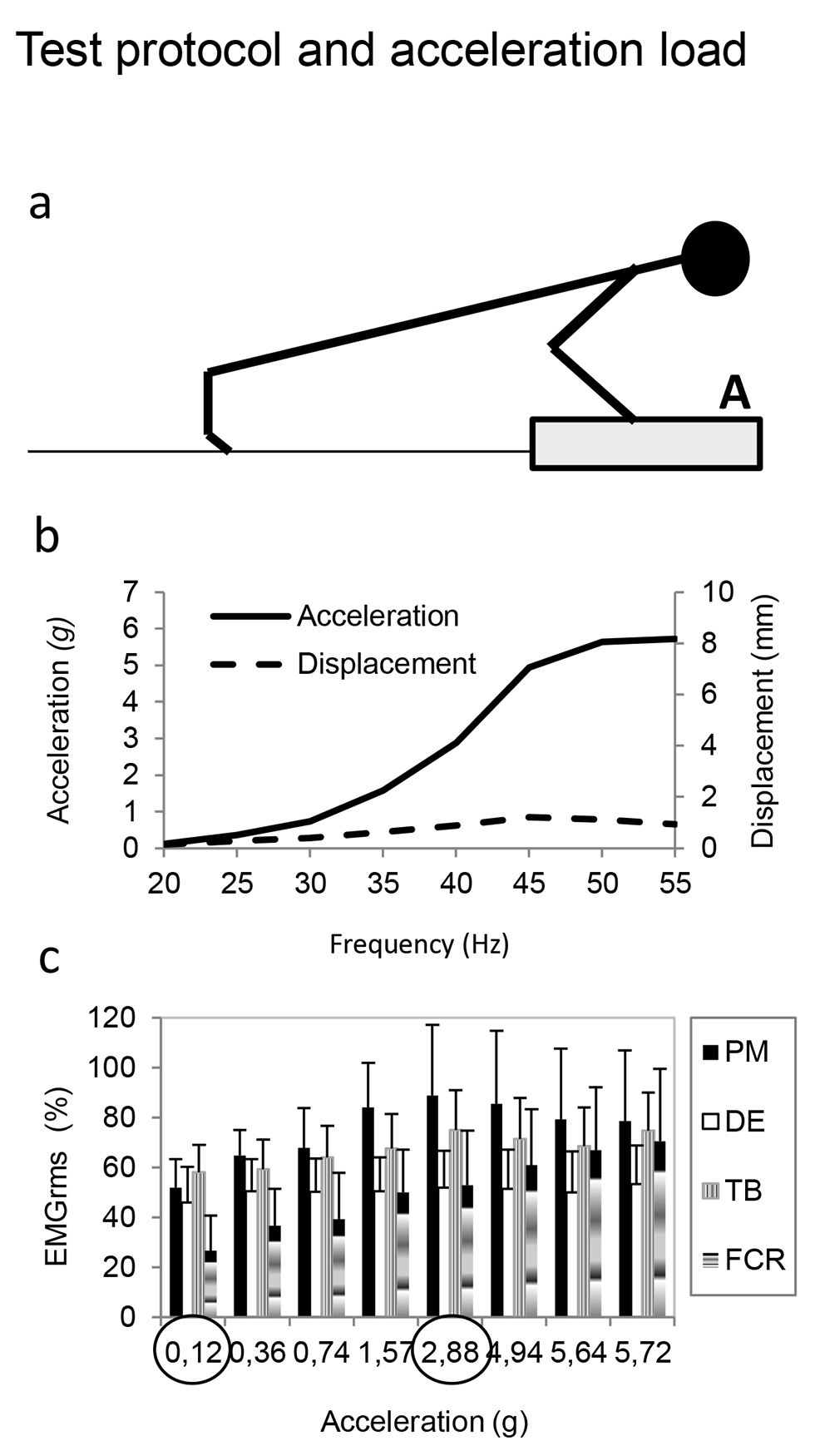

Supplement: Figure S2 — a) Position assumed by the subjects on the vibrating platform (A). b) The peak acceleration and vibrating plate displacement values were measured as the vibration frequency was increased by 5 Hz every 5 s from 20 to 55 Hz. The acceleration load values ranged from 0.1 to 5.7 g (expressed as a multiple of standard gravity, where 1 g is equal to 9.81 m·s−2). The displacement was calculated and ranged from 0.15 to 1.21 mm. c) The normalised EMGrms values (mean and SE) of the participants, which were recorded during WBV at different acceleration loads. The circles indicate the acceleration loads selected for the LVG (0.12 g) and the HVG (2.88 g). (TIF) [file pone.0111521.s002.tif]
