# Supplementary material for: Hormonal and Neuromuscular Responses to Mechanical Vibration Applied to Upper Extremity Muscles
Source: PLoS One. 2014 Nov 4;9(11):e111521. doi: 10.1371/journal.pone.0111521 (PMC4219718; doi:10.1371/journal.pone.0111521)
Supplement: Table S1 — Characteristics of the vibrational intervention. (DOCX) [file pone.0111521.s003.docx]

**Table S1**. Characteristics of the vibrational intervention.

| **Interventions** | **HVG**  **(n=10)** | **LVG**  **(n=10)** | | **CG**  **(n=10)** | |
| --- | --- | --- | --- | --- | --- |
|  |  | |  | |  |
| *Exercise type* | Isometric push-up + WBV | | Isometric push-up + WBV | | **Isometric push-up |
| *Number of repetitions (n)* | 20 | | 20 | | 20 |
| *Number of series (n)* | 2 | | 2 | | 2 |
| *Duration of each repetitions (s)* | 10 | | 10 | | 10 |
| *Rest period between repetitions (s)* | 10 | | 10 | | 10 |
| *Rest period between series (min)* | 4 | | 4 | | 4 |
| *Vibration displacement (mm)* | ~0.9 | | ~0.2 | | 0 |
| *Vibration frequency (Hz)* | 40 | | 20 | | 0 |
| *Acceleration Peak (g)* | *from 2.88 to 5.72 | | *from 0.12 to 0.36 | | 0 |

*The accelerations correspond to the highest (HVG) and to the lowest (LVG) EMGrms activity. ^**^The CG participants postured themselves on the vibration platform in exactly the same body position but without WBV.
