# Supplementary material for: Hormonal and Neuromuscular Responses to Mechanical Vibration Applied to Upper Extremity Muscles
Source: PLoS One. 2014 Nov 4;9(11):e111521. doi: 10.1371/journal.pone.0111521 (PMC4219718; doi:10.1371/journal.pone.0111521)
Supplement: Materials S1 — Experimental protocol for acceleration load determinations. (DOCX) [file pone.0111521.s004.docx]

**Materials S1. Experimental protocol for acceleration load determinations**

The subjects leaned on the platform while placing their hands palm-down and shoulder-width apart in the prone position on the upper plate of the vibration device (i.e., the isometric push-up position) (Figure S2). The subjects’ elbow joints were in a neutral position and flexed at approximately 90° and were monitored using an electrogoniometer (SG 150-Biometrics Ltd, Newport, UK). Additionally, their bodies were straight, and their feet were positioned close together to ensure that their bodies leaned on their hands and the balls of their feet.

The vertical acceleration component of the vibrating plate was measured using an accelerometer (Type ET-Acc-02, Ergotest-Innovation, Porsgrunn, Norway) according to a progressive incremental protocol as the frequency of vibration was increased by 5 Hz every 5 s from 20 to 55 Hz (Figure S2).

For the experimental protocol for acceleration load determination, all participants postured themselves in an isometric push-up position during 9 trials in the following conditions: no vibrations, and randomly 0.12, 0.36, 0.74, 1.57, 2.88, 4.94, 5.64 and 5.72 *g* (expressed as a multiple of standard gravity, where 1 *g* is equal to 9.81 m·s^-2^). There was a 1-min pause between trials, and each trial lasted 10 s. To maintain consistent positioning of the hands across the trials, the appropriate hand positions were marked on the platform. The surface EMG root mean square (EMG_rms_) for the PM, DE, TB, and FCR muscles were recorded during the trials and normalised to the MVC in each participant (Figure S2). The PM, TB, and FCR muscle activation levels were dependent on the acceleration load (P = 0.0001), whereas the DE muscle was independent of this parameter (P = 0.063). Comparative analysis of the PM muscle revealed that the EMG peaked at 2.88 *g*, and significant differences were detected at 0.12, 0.36, and 0.74 *g* (P = 0.0001). The peak EMG was maintained until 4.94 *g*, and the differences at 0.12, 0.36, and 0.74 *g* (P = 0.0001, P = 0.001, and P = 0.001, respectively) remained statistically significant. The TB muscle displayed a similar pattern in which the EMG peak was reached at 2.88 *g* (significant differences were detected at 0.12 and 0.36 *g*; P = 0.001) and was maintained until 5.72 *g* (P = 0.001). Although the FCR muscle peaked at 5.72 *g*, significant differences were been detected between 2.88 *g* and 0.12, 0.36, and 0.74 *g* (P = 0.0001, P = 0.001, and P = 0.001, respectively). Additionally, no significant differences were detected between 5.72 *g* and 2.88 or between 4.94 and 5.64 *g* (P > 0.05).

The acceleration load selected for LVG was 0.12 *g* and for HVG was 2.88 *g* because of the corresponding EMG_rms_ was respectively the lowest and the highest.

The intra-day procedure reliabilities were 0.97, 0.96, 0.95, and 0.94 for the PM, DE, TB, and FCR muscles, respectively. The inter-day reliabilities were 0.97, 0.85, 0.85, and 0.78 for the PM, DE, TB, and FCR muscles, respectively.
